# Left Ventricular Thrombus as a Complication of Clozapine-Induced Cardiomyopathy: A Case Report and Brief Literature Review

**DOI:** 10.1155/2015/835952

**Published:** 2015-11-18

**Authors:** Shahbaz A. Malik, Sarah Malik, Taylor F. Dowsley, Balwinder Singh

**Affiliations:** ^1^Department of Internal Medicine, University of North Dakota School of Medicine and Health Sciences, Fargo, ND 58102, USA; ^2^Department of Cardiology, Sanford Health, Fargo, ND 58102, USA; ^3^Department of Psychiatry and Behavioral Science, University of North Dakota School of Medicine and Health Sciences, Fargo, ND 58102, USA

## Abstract

A 48-year-old male with history of schizoaffective disorder on clozapine presented with chest pain, dyspnea, and new left bundle branch block. He underwent coronary angiography, which revealed no atherosclerosis. The patient's workup was unrevealing for a cause for the cardiomyopathy and thus it was thought that clozapine was the offending agent. The patient was taken off clozapine and started on guideline directed heart failure therapy. During the course of hospitalization, he was also discovered to have a left ventricular (LV) thrombus for which he received anticoagulation. To our knowledge, this is the first case report of clozapine-induced cardiomyopathy complicated by a LV thrombus.

## 1. Introduction

Clozapine is the most effective antipsychotic agent available for use in treatment resistant schizophrenia [1]. Despite its efficacy, the drug has been associated with serious adverse effects such as fatal agranulocytosis and cardiovascular complications (such as myocarditis and dilated cardiomyopathy) [1]. The former is monitored with the help of regular laboratory testing and by enrolling patients in the clozapine registry. However, the cardiovascular side effects still elude early detection. We present this case of dilated, nonischemic cardiomyopathy found in a patient taking clozapine to help bring this potential and gravely morbid complication to light, hopefully, increasing awareness among practitioners.

## 2. Case Presentation

A 48-year-old Caucasian male presented to the emergency department (ED) via local ambulance service, with complaints of new onset chest pain and shortness of breath with activity for past two weeks. The chest pain was present all over the chest, described as a “heavy sensation,” and had significantly improved by the time he arrived to the ED with some residual “achiness.” The pain was nonpleuritic and did not vary with postural changes. He denied any fevers, chills, cough, hemoptysis, calf tenderness, or leg swelling. He had no history of recent viral illnesses, infections, or long distance travel and no family history for premature coronary heart disease or sudden cardiac death. He had no prior reported history of coronary artery disease (CAD). He did have a history of schizoaffective disorder for which he was on aripiprazole (15 mg daily), lamotrigine (200 mg daily), benztropine (1 mg 3 times a day), and clozapine (100 mg twice a day). He had no history of smoking or recreational drug use.

History at the time of presentation was limited due to patient's psychiatric condition, as the patient would answer questions in a bizarre fashion.

His vitals in the ED revealed a heart rate of 101 beats/min, blood pressure of 95/58 mmHg, and weight of 160 lbs, and saturation was 95% on room air. Positive findings on physical examination were elevated jugular venous pulsations, fine crackles at bilateral lung bases. Cardiovascular exam revealed a regular rhythm, elevated rate, and normal heart tones without any obvious S3. Pulses were palpable and symmetrical in bilateral upper and lower extremities with no peripheral edema. The patient was alert, oriented to self and time, only. On mental status examination, his thought process was tangential.

A 12-lead electrocardiogram showed sinus tachycardia of 103 beats/min and a new onset left bundle branch block (LBBB), with prolonged corrected QT interval (QTc) of 497 ms; there were no ST segment or T-wave changes. Point-of-care troponin and subsequent cardiac troponin I were both within normal range. Remaining laboratory and biochemical findings are reported in the Table 1.

Chest X-ray showed significant cardiomegaly along with prominent pulmonary vascular markings consistent with pulmonary edema. Due to his complaints of chest pain and shortness of breath along with finding of new LBBB, the patient was taken immediately to the cardiac catheterization laboratory and loaded with aspirin en route. Coronary angiography revealed an essentially normal coronary anatomy with no significant lesions or evidence of occlusive CAD. Ramus intermedius was identified. Left ventricular angiogram showed an ejection fraction (EF) of 15%. Left ventricle end diastolic pressure (LVEDP) was significantly elevated at 35 mm Hg (normal 6–12 mm Hg).

The patient was admitted to the intensive care unit (ICU) and initiated on dobutamine at 5 mcg/kg body weight/min for inotropic support. Transthoracic echocardiogram (TTE) revealed an EF of 10% with severe diffuse hypokinesis, normal left ventricular wall thickness, with evidence of elevated left atrial pressures along with markedly dilated left and right atria. Mild-to-moderate mitral regurgitation was reported. Pulmonary artery systolic pressure was elevated at 69 mm Hg. No pericardial effusion was identified. Inferior vena cava (IVC) was markedly dilated as well, showing <50% variation with breathing.  Table 2 shows the results from the multiple echocardiography measurements during hospitalization.

He was initially started on diuresis with intravenous (IV) furosemide 40 mg daily that was increased to twice daily the next day. With diuresis the patient's weight gradually came down to 153 lbs (nadir at the time of discharge) and he improved symptomatically. On day 3, he was switched to per oral (PO) furosemide 40 mg daily. He initially required vasopressor support while being diuresed but was later weaned off it on day 6. Furosemide was decreased to 20 mg PO daily at that point and he was initiated on low dose metoprolol succinate at 12.5 mg daily. Lisinopril 2.5 mg daily was added on day 7. Metoprolol was increased to 25 mg daily on day 9, but lisinopril dose could not be increased further on account of blood pressures remaining in the low-normal range. Repeat TTE did not reveal any change in EF. His vitals stabilized and he started ambulating with physical therapy.

Given the relatively young age of the patient, absence of viral prodromal symptoms, unremarkable coronary angiogram excluding an ischemic etiology, normal cardiac enzymes excluding active cardiac myonecrosis, iron studies not suggestive of any iron overload condition to explain the patient's dilated cardiomyopathy, and absence of other risk factors to explain the cause for the profoundly low EF, clozapine was thought to be possible culprit of the cardiomyopathy.

Review of outside medical records indicated that the patient was on clozapine due to “command hallucinations to harm himself.” Going through the clozapine registry, it was ascertained that the patient had been on 300 mg a day of clozapine (200 mg at bedtime and 100 mg in the morning) since June 2010. For unknown reasons, the dose was decreased to 200 mg a day (100 mg in the morning, 100 mg at bedtime) a few weeks prior to hospitalization. Consult liaison (CL) psychiatry team was consulted to assist in treatment planning. Given the possibility of clozapine-induced cardiomyopathy CL team tapered and discontinued clozapine. On day 7, aripiprazole was increased to 30 mg daily (admitted on 15 mg daily); lamotrigine was continued at 200 mg daily. Benztropine was later discontinued. On day 10, he was initiated on PO olanzapine 5 mg at bedtime and titrated to 10 mg on day 15.

Kidney function and hematological laboratory values remained essentially unchanged throughout the course of the hospitalization. No cardiac arrhythmias were observed during the stay. Cardiac rehabilitation was initiated during hospitalization; his functional status gradually improved during stay. Repeat TTE at three weeks showed an EF of approximately 10% but now with a new large, echogenic, mobile, mass which measured 26 mm × 20 mm on the basal inferoseptal wall. This was representative of a LV mural thrombus. The patient's mitral regurgitation also looked worse, based on echocardiographic assessment. He was immediately started on parenteral anticoagulation with low molecular weight heparin (enoxaparin) 80 mg twice daily and then started on oral warfarin. Parenteral anticoagulation was continued until the international normalized ratio (INR) level reached a therapeutic range of greater than 2.

Cardiothoracic surgery was consulted in order to obtain an opinion in regard to the patient's mitral regurgitation. The risks of surgery for mitral valve repair outweighed the benefit in their opinion and thus no surgery was recommended. A repeat TTE on day 33 showed the size of the LV thrombus had decreased to approximately 19 mm × 13 mm (Table 2). Mitral regurgitation remained the same. During all this time, his vitals remained stable.

## 3. Outcome and Follow-Up

The patient improved clinically and by the end of the hospitalization course, his dyspnea improved and he was able to walk over thousand feet with cardiac rehabilitation and physical therapy. He was discharged on day 37, on metoprolol succinate at 25 mg daily, lisinopril 2.5 mg daily, and warfarin. Furosemide was switched to torsemide 20 mg daily on the day of discharge. In terms of the patient's psychiatric medications, he was sent home on aripiprazole 30 mg daily, lamotrigine 200 mg daily, and olanzapine 10 mg. He was scheduled to follow-up with an outpatient primary care provider, cardiologist, and a psychiatrist on discharge. The patient continues to follow with cardiology regularly with not much improvement in ejection fraction at three months of follow-up. This has prompted consideration for cardiac resynchronization therapy, in the near future.

## 4. Discussion

In this report, we described a case of a young male with schizoaffective disorder with no prior history of coronary heart disease presenting with a new onset heart failure and cardiomyopathy. Hypertensive heart disease and genetic and toxic etiologies were ruled out on the basis of the patient's medical history and physical examination. History was also negative for the use of chemotherapeutic agents (notably anthracyclines) or other medications such as antiretroviral drugs, phenothiazines, or chloroquine that could have led to the development of cardiomyopathy. Ischemic cause was excluded based on coronary angiography, whereas infectious and metabolic etiologies were ruled out based on laboratory analyses. In the absence of any other obvious cause for the patient's cardiomyopathy, clozapine was considered the culprit and the medication was discontinued. The patient's case was complicated by a LV thrombus. LV thrombi are an uncommon yet known complication of anterior wall transmural myocardial infarctions (10%) [2]. Furthermore, severe mitral regurgitation has been thought to have a protective role in LV thrombus formation in patients with reduced ejection fraction [3]. Thus, to our knowledge, this is the first case report of cardiomyopathy related to clozapine that was further complicated by an LV thrombus, despite presence of mitral regurgitation. It is unclear whether clozapine itself could be implicated in the thrombus formation, as there have been reports of possible association of clozapine with venous thromboembolic phenomenon [4]. However, it is just as likely that the thrombus was related to poor LV function and the cardiomyopathy itself.

Schizophrenia is currently understood as a psychiatric illness with progressive clinical, neuropsychological, neurophysiological, and neurostructural deterioration [5]. It typically involves recurrent or chronic psychosis. It has been ranked by the World Health Organization as one of the top ten illnesses contributing to the global burden of disease [6]. Approximately 1% of the world's adult population suffers from schizophrenia [7]. Clozapine is the treatment of choice for patients with treatment resistant schizophrenia [8–10]. For patients with schizophrenia, it is the only antipsychotic agent with a demonstrated significant reduction in suicidality and it usually produces few or no extrapyramidal symptoms (such as tardive dyskinesia or dystonia caused by typical antipsychotics) [4, 11]. Despite its efficacy, the drug has been associated with serious adverse effects such as fatal agranulocytosis and toxic megacolon and cardiovascular complications including myocarditis and dilated cardiomyopathy. As a result, it is not considered a first-line treatment and is reserved for patients with treatment resistant schizophrenia/schizoaffective disorder [12–14].

Characteristics of patients with clozapine-induced cardiomyopathy were detailed in a systematic review from 2014, which included reviewing data from 26 cases of clozapine-induced cardiomyopathy [15]. They reported a mean age of 33.5 years, a mean dose of 360 mg, and average time to symptoms onset of 14.4 months and most commonly reported echocardiographic findings as being reduced ejection fraction with global dysfunction [15]. Specific mention of dilated cardiomyopathy was in 39% of individual case reports per the systematic review [15]. Approximately 80% of clozapine treated patients in whom cardiomyopathy was reported were less than 50 years of age, according to a manufacturer adverse event database [16].

The incidence of dilated cardiomyopathy in the general population has been reported to be 7.5–10.0 per 100,000 population [17]. A recent cohort study performed in Australia describes the incidence of clozapine-induced myocarditis and cardiomyopathy to be 3.88% and 4.65% (or 2.26 per 100 patient years), respectively [18]. The rate of cardiomyopathy in clozapine treated patients in the US was shown to be 8.9 per 100,000 person-years according to national databases reporting adverse drug effects [19]. On the other hand, the incidence of clinically severe clozapine-induced cardiomyopathy was reported as 51.5 per 100,000 patient-years [20]. Time to onset of clozapine-induced cardiomyopathy has varied between reports. One case report noted it at 3 weeks [21]. Another case report detailed the discovery on postmortem exam after the patient had been on clozapine for 4 years [22]. A case series described three men developing symptoms secondary to severe left ventricular dysfunction from clozapine use on average one year before diagnosis [23]. The clinical manifestations of clozapine-induced cardiomyopathy range from subclinical presentation [24] to fulminant pulmonary edema and cardiogenic shock [25, 26]. Common presenting symptoms include shortness of breath, palpitations, cough, fatigue, chest pain, and sometimes atypical symptoms such as worsening psychiatric mental status [27, 28]. Diagnosis of clozapine-induced cardiomyopathy has typically been on clinical exam, electrocardiography, and echocardiography. There are two reports in literature detailing the diagnosis on postmortem examination [22, 29].

Mechanism of association of clozapine with cardiomyopathy is still elusive and lacks consensus in the literature. Cardiomyopathy is not as closely related to other antipsychotics as it is to clozapine [20]. One hypothesis suggests that there may be a direct toxicity similar to anthracycline-induced cardiomyopathy [25]. Second explanation is that cardiomyopathy may evolve from clozapine associated myocarditis. Some authors have suggested that exposure to prior antipsychotics, illicit drugs, or alcohol may plays a factor [13].

Treatment of clozapine-induced cardiomyopathy involves cessation of the drug [15]. Guideline directed therapy for heart failure should be instituted [15]. Other treatment goals include prevention of additional cardiac injury related to recreational drug such as alcohol and amphetamines. Alternative antipsychotics such as olanzapine have been used in most other cases [15]. Several reports have noted that there was an improvement in cardiac function on echocardiogram after cessation of clozapine [15, 20]. However, an interesting case report suggested efficacy of beta blockers in association with an angiotensin-converting enzyme inhibitor to decrease the risk of cardiac deterioration and possibility of resuming drug in patients with psychiatric symptoms refractory to other antipsychotics [30]. In general, patients with an ejection fraction of <25% at the time of diagnosis have a poor prognosis including the highest risk of mortality with limited recovery [15]. Patients with an ejection fraction of 25–40% generally show significant improvement [15]. Patients with an ejection fraction of >40% usually show near complete recovery of cardiac function at 6 months, after cessation of clozapine and with normal heart failure treatment [15]. It should be noted that one study demonstrated that 80% of patients withdrawn from clozapine for medical reasons developed a psychotic relapse [31]. Therefore, clozapine cessation should be done under supervision of a psychiatrist and appropriate alternative medication should be substituted to prevent relapse.

## Figures and Tables

**Table 1:** Laboratory and biochemical analyses.

Laboratory parameters (units)	Patient's results (reference range)
Complete blood count	
White cell count (K/*μ*L)	8.2 (4–11)
Hemoglobin (g/dL)	13.1 (13.5–17.5)
Hematocrit (%)	38.3 (40–50)
MCV (fL)	83.6 (80–98)
Platelet count (K/*μ*L)	175 (normal 140–400)
Chemistry	
Serum glucose (mg/dL)	126 (70–100)
Sodium (mEq/L)	140 (135–145)
Potassium (mEq/L)	3.5 (3.5–5.3)
Chloride (mEq/L)	104 (99–110)
Magnesium (mg/dL)	1.9 (1.8–2.4)
BUN (mg/dL)	16 (6–22)
Creatinine (mg/dL)	1.2 (0.8–1.3)
Total bilirubin (mg/dL)	1.9 (0.2–1.2)
Alanine aminotransferase (AST) (U/L)	6 (0–55)
Aspartate aminotransferase (AST) (U/L)	12 (0–35)
Alkaline phosphatase (U/L)	73 (30–150)
Iron studies	
Total iron (mcg/dL)	30 (65–175)
Iron saturation (%)	10 (20–50)
Ferritin (ng/mL)	88 (21–275)
Total iron binding capacity (mcg/dL)	307 (250–400)
Cardiac markers	
Troponin (ng/mL)	0.01 (0.00–0.08)
Troponin I (ng/mL)	0.019 (0.00–0.028)
Creatine kinase (CK) (U/L)	48 (30–200)

**Table 2:** Echocardiography measurements during hospitalization demonstrating left ventricle dysfunction.

Parameter	Day 1	Day 6	Day 14	Day 21	Day 33
Left ventricular ejection fraction (%)	10	10	10	10	10
Left ventricular internal diameter (end diastolic) mm (millimeter)	82	81	79	73	73
Left ventricular internal diameter (end systolic) mm (millimeter)	75	70	73.8	70	68
Peak E/A ratio (early-to-late ventricular filling ratio of mitral flow)	1.66	—	—	—	—
Deceleration time (of mitral E wave) ms (meters per second)	124	—	—	—	—
Pulmonary artery peak systolic pressure in millimeter mercury	69	65	57	64	72
Left ventricle thrombus in millimeter × millimeter	—	—		26 × 20	19 × 13
